# Governing Migration through Multi‐Level Governance? City Networks in Europe and the United States^*^

**DOI:** 10.1111/jcms.13214

**Published:** 2021-06-22

**Authors:** Tiziana Caponio

**Affiliations:** ^1^ University of Turin Turin; ^2^ European University Institute Florence

**Keywords:** multi‐level governance, city networks, migration policy, EU and US comparison

## Abstract

City networks (CNs) are often enthusiastically regarded as key actors in processes of Europeanization and multi‐level governance (MLG) policy‐making in Europe and beyond. However, systematic research on highly contentious issues like migration is still scarce. Building on an understanding of MLG as a specific mode or instance of policy‐making, in this article I seek to understand why and how CNs engage in MLG‐like policy‐making on a typical issue of state sovereignty. I apply the causal process‐tracing method to analyse the genesis and policy actions undertaken in the last two decades by two migration CNs in different multi‐level political settings: the Eurocities Working Group on Migration and Integration (WGM&I) in the EU and Welcoming America (WA) in the US. The results show that, notwithstanding the differences in the institutional settings, in both contexts instances of MLG policy‐making have taken place in the shadow of the will of the national governments, which remain fundamental gate‐keepers even in the EU supranational polity, where the European Commission has been particularly active in supporting migration CNs' initiatives.

## Introduction

In the last two decades, city networks (CNs) have started to be regarded as new actors in the governance of globalization, linking cities across nation states and with supranational governance institutions (Barber, 2013; Agranoff, 2018, p. 217). These arguments resonate with the burgeoning debate on the role of subnational actors in Europeanization processes (Nadalutti, 2013; Huggins, 2018) and European multi‐level governance in such different policy fields as climate change mitigation (Kern and Bulkeley, 2009), urban regeneration policy (Payre, 2010) and more recently migration (Penninx, 2015).

However, empirical research on the nexus between city networking and multi‐level governance (MLG) on highly contentious issues like migration remains scarce. Building on an understanding of MLG as a specific mode or instance of policy‐making (Alcantara *et al*., 2016), in this article I seek to understand why and how CNs engage in MLG‐like policy‐making arrangements on the contentious migration issue. Migration policy can be considered a least‐likely case for MLG since states have always been reluctant to share power in this area with either local or supra‐national authorities (Boswell and Geddes, 2011). Nevertheless, migration CNs have been established in various multi‐level political systems (Filomeno, 2017). An in‐depth analysis of the multi‐level political dynamics around migration policy therefore appears to be of the utmost importance in order to push forward theorization on the CNs‐MLG nexus and to broaden our understanding of processes of state authority reconfiguration on such a key state sovereignty issue.

To this end, I take an original transatlantic perspective and apply causal process‐tracing to explore the factors and mechanisms accounting for the genesis and involvement in MLG policy‐making of two migration CNs: the Eurocities Working Group on Migration and Integration (WGM&I) in the EU supranational system and Welcoming America (WA) in the US federal state. The results show that, notwithstanding the differences in the institutional settings, problem pressure and CN leaders' agendas, in both contexts instances of MLG policy‐making have taken place in the shadow of the will of the national government. Certainly, CNs have been able to affirm the crucial role of local authorities in making everyday migration policy (Oomen, 2020), yet national governments' remain fundamental gate‐keepers even in the supranational EU multi‐level polity, where the European Commission has been particularly active in supporting initiatives on migration CNs (Penninx, 2015).

The paper is structured as follows. In the next section, after a discussion of the still scarce literature on the CN‐MLG nexus in migration policy, I propose an analytical framework to make sense of CNs engagement in MLG policy‐making in different multi‐level political systems. In the second section, I present the empirical study and provide details of the case selection and methodology. The third and fourth sections provide structured narrative assessments of how the two analysed migration CNs have been involved in MLG policy‐making from their foundation until 2019 in the EU and the US. In section five, I discuss the research findings on the factors and mechanisms underlying the CN‐MLG nexus. The article concludes by elaborating on the implications of my research for theorization on the re‐definition of state authority vis‐à‐vis contentious globalization challenges like migration.

## I MLG and City Networks. Exploring the Nexus

Since the late 1990s, city networking has attracted the attention of scholars in different disciplines, leading to a lively debate on the mobilization of cities on the global scene (see, for example, Aldecoa and Keating, 1999; Acuto and Rayner, 2016; Herrschell and Newman, 2017). In Europe, political scientists have linked the flourishing of CNs to the reform of the EC structural funds (1988–93) and the adoption of the Maastricht Treaty in 1992 (Hooghe and Marks, 2001). Following in these footsteps, scholars working on climate change mitigation (Gustavsson *et al*., 2009; Kern and Bulkeley, 2009) and more recently on migration (Penninx, 2015; Caponio, 2018; Scholten *et al*., 2018) have emphasized the nexus between CNs and MLG.

CNs have been conceptualized as organizations that bring together local authorities on a voluntary basis in order to pursue some kind of collective interest or purpose (Huggins, 2018). Since CNs lack authoritative power, to carry out their initiatives they have to rely on the kind of collaborative relationships that scholars attribute to MLG policy‐making (Bache and Flinders, 2004), that is, vertical co‐operation with higher tiers of government on the one hand and horizontal collaboration with key stakeholders and non‐governmental civil society actors on the other.

However, to make sense of the CN‐MLG nexus, especially on under‐researched topics in the MLG literature and highly politicised issues like migration, clarification of the still elusive notion of MLG (Tortola, 2017) is needed. Introduced by Gary Marks (1993) to account for the European Community as ‘a system of continuous negotiations among nested governments at several territorial tiers – supranational, national, regional and local’ (p. 392), over the course of time the concept of MLG has been undergoing a process of semantic expansion. On the one hand, MLG has continued to represent a key theoretical and analytical perspective in scholarly debates on the *sui generis* EU polity (for recent reviews, see Stephenson, 2013; McNamara, 2018); on the other, MLG has become a metaphor to capture processes of dispersion of state authority more generally ‘across interdependent, and yet autonomous, public authorities and non‐public organizations placed at different levels of government’ (Hooghe and Marks, 2001, p. xi; see also Hooghe and Marks, 2000). According to critics, however, such an expanded definition runs into concept indeterminacy, with the result that ‘any complex and multi‐faceted political process can be referred to as multi‐level governance’ (Peters and Pierre, 2004, p. 88).

To address this criticism, public policy scholars have suggested a strategy of ‘concept shrinking’ and defined MLG as a specific *instance* or configuration of policy‐making within the broader category of multi‐level politics (Scholten, 2013; Alcantara et al., 2016; Caponio and Jones‐Correa, 2018). Three features appear to underlie such definitions: (1) different levels of government are simultaneously involved; (2) non‐governmental actors at different levels are also involved; and (3) the relationships defy existing hierarchies and take the form of non‐hierarchical networks based on co‐operation and consensus building (see also Piattoni, 2010).

**Figure 1:**
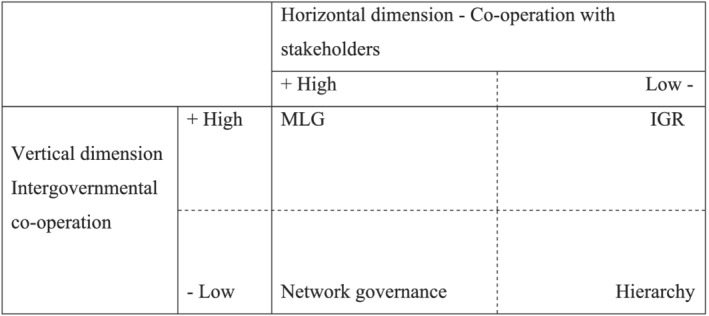
Configurations of Policy‐Making in Multi‐level Political Settings. A local Perspective.

This article builds on an understanding of MLG as a specific instance of policy‐making and aims to understand how and to what extent processes of city networking contribute to re‐defining traditional state‐based hierarchical modes of decision‐making on a matter typically in the realm of national sovereignty – that is, migration. To this end, in Figure 1 I propose a conceptual space of possible configurations of policy‐making in multi‐level political settings defined by the intersection of two continua representing levels of collaboration on the vertical and horizontal dimensions. MLG as an instance of policy‐making is characterized by co‐operative relations on both dimensions, and it coexists with other instances of policy‐making – that is, hierarchy, intergovernmental relations and networked governance.

More specifically, hierarchical modes of policy‐making in which subnational authorities have to comply with directives from above to implement national policy are characterized by power and top‐down co‐ordination. This top‐down mode of regulation has for a long‐time been assumed to be the very hallmark of the migration policy field because of the sensitive state sovereignty issues involved such as border controls and national identity (Boswell and Geddes, 2011). However, since the late 1990s, scholars have started to notice states attempting to shift responsibilities up to international and supra‐national institutions, out to non‐public actors and down to local‐level authorities (Guiraudon and Lahav, 2000). Complex multi‐level political dynamics have become a matter of increasing attention in scholarly literature (for a review concerning Europe and the US, see Filomeno, 2017).

Along with MLG and hierarchy as defined above, Figure 1 identifies two more modes of multi‐level politics – that is, intergovernmental co‐operation and network governance. In intergovernmental co‐operation, collaboration only takes place on the vertical dimension, somewhat reflecting the mode of operation of so‐called ‘migration co‐operative federalism’ (Spiro, 2001). This model can also be found in unitary systems when intergovernmental platforms or venues are promoted on specific issues. In the bottom left of Figure 1 we find modes of policy‐making of a network‐governance type (Klijn and Koppenjan, 2016) based on building collaborative interactions between public, private and societal actors, thereby challenging the separation between public and non‐public actors in decision‐making and/or in the implementation of a specific policy.

The four modes of policy‐making identified above should be understood as ideal types –that is, pure models against which concrete cases can be assessed. In this conceptual map, CNs are likely to be found in different positions depending on the policy‐making relations they are engaged in. This is consistent with the results of several empirical studies showing that CNs can perform different functions (Kern and Bulkeley, 2009; Caponio, 2018; Oomen, 2020) and that MLG is just one possible policy‐making configuration and not necessarily the most relevant one (Scholten, 2013). The question then arises of which factors and mechanisms account for the engagement of CNs in instances of MLG policy‐making in the contentious migration policy field.

Research on MLG in Europe has regarded CNs as the result of bottom‐up mobilization by local authorities to engage in EU policy‐making beyond traditional government structures (Benz *et al*., 2015). Regarding migration policy, Penninx (2015) notices the importance of the dialogue structures and funding provided by the European Commission (EC) in stimulating the organization of transnational networks of municipalities (p. 107). In other words, the CN‐MLG nexus is understood as an outcome of the particular institutional opportunity structure characterizing the EU polity.

However, other authors (see, for example, Agranoff, 2018) emphasize that because of the interdependences created by globalization in migration as in other policy fields, local governments play a key role in co‐ordinating complex systems of relations with other levels of government and with multiple non‐government actors (p. 143). Hence, migration CNs reflect the increasing centrality of local governments in ensuring effective policy implementation through MLG policy arrangements. This development underlies migration policy‐making in different regions of the world (primarily Europe and North America) and also at the global level, as, for instance, is indicated by the Mayors Migration Council established in December 2018 (Oomen, 2020).

However, in most accounts taking either an EU‐centred institutionalist or a macro‐structural globalization perspective, there is often a lack of clear understanding of how EU institutions and new global challenges can trigger CNs' engagement in MLG policy‐making. Studies taking a more agent‐centred perspective seem to indicate that leadership matters. In particular, Scholten *et al*. (2018) highlight the entrepreneurial role of the aldermen of the main Dutch cities in establishing structures for institutional multi‐level co‐operation with the national government and the EC on the locally salient issue of EEC citizens' access to welfare services. Other scholars adopting an organizational perspective (see, for example, Payre, 2010 on Eurocities), however, contend that it is CN staff officers who take the lead in decisions on the type of policy‐making relations to form with other public and non‐public actors. Hence, for micro‐level and agent‐based approaches, differences in the engagement of CNs in MLG policy‐making are likely to depend either on the strategies of CN political leaders or on the policy agenda pursued by internal staff officers.

However, leaders' strategies are not formed in a void but are likely to be deeply influenced by the institutional structures and/or the sense of urgency underlying specific issues. Hence, we can conceptualize CN leaders' views and agendas as one possible mechanism linking specific factors, that is the institutional structure and/or new policy challenge, to MLG policy‐making. Other factors and mechanisms are likely to be also at play. This article aims exactly to unravel such factors and mechanisms to better understand why and how CNs get engaged in MLG‐like policy‐making arrangements on the contentious migration issue. Such an empirical assessment, I posit, is key to understanding the promises and limits of the concept of MLG in making sense of state authority reconfiguration in the EU and beyond.

## II Methodological Note

To throw light on the nexus between CNs and MLG policy‐making on the highly salient migration issue, I adopt a research strategy based on a combination of process‐tracing and comparative case study. According to Kay and Baker (2015), such a strategy is particularly well‐suited to dealing with the theoretical pluralism characterizing research on public policy. On the one hand, it enables a comparative assessment of middle‐range theoretical propositions; on the other, it allows for identification of other possible explanatory mechanisms and factors not considered in the literature.

Hence, in this study I do not follow a strictly hypothesis‐testing path but, as Beach and Pedersen (2019, p. 10) suggest, I use theory‐building process‐tracing, which is defined as ‘a creative iterative process of moving back and forth between empirical probing and theorisation in which inference is based on cross‐case comparison of within‐case causal mechanisms and configurations of factors’. As will be clear, case selection is of paramount importance to trace in‐depth the causal factors and mechanism(s) linking X to Y – that is, in our case CNs to MLG policy‐making.

To this end, on the basis of a preliminary screening of internet websites, policy reports and informal exchanges with experts, I identified two ‘typical cases’ (Beach and Pedersen, 2019, p. 11), that is migration CNs that, notwithstanding all the differences between the EU and US multi‐level political systems, have been similarly involved in processes of MLG‐like policy‐making. The cases are the Eurocities Working Group on Migration and Integration (WGM&I) in the EU supranational context and Welcoming America (WA) in the US federal state. Table 1 provides some general information on the cases selected. Notably, whereas Eurocities WGM&I is a pure case of a ‘transnational municipal network’ (Kern and Bulkeley, 2009) – that is, an organization formed by municipal governments in different countries, WA presents a more varied and grassroots‐based – NGOs, civil rights associations, and so on – membership, even though city/local governments constitute the core constituency.

**Table 1:** Main Features of WGM&I and WA

	*Year of establishment*	*Membership*	*No. of cities*	*Access*
WGM&I	2004 (2001)	City governments and metropolitan areas	78	Eurocities annual fee, ranging between €21,450 (full member*) and €4,420 (associate partner)
WA	2009 (2001)	Local governments (cities and counties), non‐profit organizations	97	Annual fee ranging between $200 (General) and $2,500 (Premium)

* Full membership allows participation in Eurocities different Forums and Working Groups, not only the WGM&I.

In‐depth case studies were carried out on each network between 2018 and 2019. The primary sources of data were the CN official documents and web pages, which were systematically downloaded in different time periods – that is, in November–December 2018 for the first time, and then in the spring and autumn of 2019 to check for new documents and updates. Overall, a corpus of 38 documents for the WGM&I and 29 documents for WA was constructed and qualitatively analysed in order to gather information on processes of involvement in MLG policy‐making. The resulting empirical narratives were then systematically triangulated with other data sources – that is, official documents downloaded from the websites of the institutions and organizations participating together with the CNs analysed in policy‐making processes on migration; qualitative interviews with key informants in the CNs (like officers and/or political leaders) and other relevant institutions and organizations (see the list of interviews); participant observation at some key events organized by the two networks in 2018–19; and informal conversations with city representatives and other stakeholders participating in these events.

In the following section, I present the research evidence in structured narratives. In particular, I follow a chronological order to reconstruct the sequence of events underlying the involvement and participation of the two CNs in instances of MLG and to identify the specific factors and mechanisms underpinning these sequences. In the comparative section I discuss the main similarities in the factors and mechanisms at work in the two cases and flesh out the broader implications of the research results for making sense of MLG policy‐making on the highly contentious migration issue.

## III Eurocities and the Working Group on Migration and Integration

In 2001, in the turbulent context of 9/11 and debates on ‘fortress Europe,’ the municipalities of Rotterdam and Barcelona started to mobilize a group of 15 cities to set up a migration observatory and carry out a first mapping of their integration policies (Council of Europe, 2003, p. 63; WGM&I_int1). However, the WGM&I was only formally established as one of the working groups of the Eurocities Forum for Social Affairs in 2005. The official documents of this period (for an extensive analysis, see Gebhardt and Güntner, 2021) reveal the ambition of the WGM&I to have a say in the definition of the ‘European Framework on Integration’ – which was introduced in 2002 in the context of the 1999 Tampere Programme – and to establish direct relations with the European Commission.

Regarding the policies of national governments, we recommend […] that in the design of policy concerning immigration and asylum, reception and integration, national governments take into account the most essential element: the impact of policy at the local level … [p. 6]. Regarding the policies of the European Union […] [w]e advise the Commission to develop a consultation framework with the large cities and their associations in Europe in order to be adequately informed of all the issues concerned and the impact of European policies at the local level (Eurocities, 2004, p. 7).

Hence, rather than MLG, the political goal of the cities taking the lead in the early days of the WGM&I – that is, Rotterdam and Barcelona – was to establish a system of collaborative inter‐governmental relations with EU institutions. As Gebhardt and Güntner (2021) report and as was confirmed by my interviewees, the people mobilized in this period were primarily city officers, while mayors like Ivo Opstelten of the city of Rotterdam played a key role in bringing the requests of the working group to the attention of the EC. This activity lobbying for more influence led to several symbolic and concrete results. At the symbolic level, in 2005 the Communication on a Common Agenda for Integration (European Commission, 2005) explicitly stated that ‘integration takes place at the local level as part of daily life and [...] [e]ngaging local communities in working together is thus crucial’(p. 3). At the more practical level, in 2006 an institutionalised partnership between Eurocities and the EC was established in Rotterdam: the so‐called Integrating Cities Process (ICP). The Milan Declaration, which was signed in 2007, formally confirmed the commitment of the two parties to ‘a constant dialogue and co‐operation for the success of immigrant integration, pursuing policies based on the principles of partnership, empowerment and good governance’ (Comune di Milano, 2007, p. 3). It also detailed the instruments of the partnership – that is, Integrating Cities conferences and a working programme co‐ordinated by Eurocities consisting of bi‐annual projects funded by the EC. According to Gebhardt and Güntner (2021, 5), ‘the series of projects that were developed through the partnership brought staff from the secretariat acting as coordinator to the fore and led to a more managerial culture in the migration work’. This was also confirmed in my interviewees: in 2008 Eurocities hired a policy advisor responsible for the area of migration and employment (Newsletter March 2008), and other project managers were recruited later (WGM&I_int1; WGM&I_int2).

With the unfolding of the so‐called ‘European refugee crisis’ between 2014 and 2015, however, two different and somewhat parallel agendas emerged: one pursued primarily by Eurocities staff officers and city officials and one by city mayors.

The first agenda appears to have been firmly anchored to the partnership with the EC and coordination of the EU‐funded project. Since 2010, efforts had been directed at implementing the so‐called Integrating Cities Charter, which codified city commitments on diversity and equality policies. Symbolic events like the Integrating Cities Conferences have always been open to various categories of actors, including international organizations like the International Organization for Migration and the United Nations High Commissioner for Refugees, international research institutes and think tanks, NGOs and immigrant rights organizations, and in some cases national governments too. However, no formal policy‐making dialogue had ever been established.

The second agenda, that of mayors and cities' political representatives, was articulated primarily through the WGM&I and presented a more confrontational approach. A clear case in point is the Eurocities Statement on Asylum in Cities (Eurocities, 2015), which was drafted on the impulse of southern European cities like Milan, Athens and Barcelona. Following in its footsteps, then Mayor of Athens George Kaminis launched a new initiative, Solidarity Cities (SC), which my interview partners described as a ‘plea’ or ‘call for action’ to provocatively advocate for a bottom‐up asylum seeker redistribution mechanism managed by cities (WGM&I_int1; WGM&I_int2; WGM&I_int6; WGM&I_int7).

Eurocities staff officers managed the potential contradictions between the two agendas through a strategy of ‘internalization’ of SC. The new network was officially presented during the Eurocities Social Affairs Forum held in Athens in October 2016 and a programme of initiatives centred on knowledge exchange, capacity building and advocacy was started.

The Mayor of Athens shed light on the work of cities in the midst of the emergency … yet from the beginning there was a need to go beyond mere criticism of national non‐policies. Eurocities was able to shape a programme of actions attracting the attention not only of mayors but also of policymakers at different levels of government (WGM&I_int2).

This policy agenda combining advocacy and policy expertise characterized the work of Eurocities in the Partnership for the Integration of Immigrants and Refugees (Partnership). The Partnership was one of the thematic fora created in the context of the Urban Agenda for the EU, which was launched in May 2016 with the Pact of Amsterdam by the Dutch presidency of the EU Council and the ministers responsible for urban matters.

The so‐called Pact of Amsterdam, other than the name might suggest, had very few relations with the city … The preparation meetings took place in Amsterdam, but the whole process was steered by our colleagues of the Ministry of the Interior, who have responsibility over urban planning‐related issues […] There were already the groups on housing, air quality and poverty working as pilot, and there was a fourth theme they [people in the Ministry] were looking at, migration and inclusion … The then mayor, Eberhard van der Laan, had this idea that Amsterdam should be a responsible capital city, and take on responsibility on international issues … and so we, as city of Amsterdam, we stepped in and offered to chair the Partnership …. Parallel to the mayor's political contacts, we approached DG Home officers and proposed them to co‐chair the Partnership with us (WGM&I_int8).

In fact, according to other interviewees (WGM&I_int4 and WGM&I_int5), in spring 2016 the mayor of Amsterdam, together with those of Barcelona, Athens, Ghent, Helsinki, Berlin, Leipzig, Malmö, Paris and Rome, met with Dimitris Avramopoulos, the Commissioner for Migration, Home Affairs and Citizenship, to denounce the failure of EU refugee redistribution and ask for direct support for cities (see also Gebhardt and Güntner, 2021).

At the beginning, the European Commission chaired by Junker was clearly not interested in cities; matters of migration were dealt with national governments only … In the midst of the crisis though, we saw a change of attitude. States were not collaborating at all and the European Commission risked remaining isolated … Cities started to be regarded again by the DG Home as allies, like before with Commissioner Frattini, when the Integrating Cities Project was launched (WGM&I_int1).

The Partnership included numerous representatives of subnational governments – that is, along with Eurocities, Solidarity Cities (and more specifically the mayors of Athens, Barcelona, Berlin and Helsinki), the Council of European Municipalities and Regions (CEMR), the European Committee of the Regions and the Urbact Programme. Other participants included representatives of EU member states (Denmark, Greece, Italy and Portugal), of the DGs Regio and Employment and Social Affairs, the Joint Research Centre, the Council of Europe Development Bank (CEB), the European Council for Refugees and Exiles (ECRE), the European Investment Bank (EIB) and the Migration Policy Group (MPG). The Partnership's action plan, the result of a year‐long decision‐making process, also engaged NGOs and migrant associations through specific work conferences.

Eurocities took responsibility for the implementation of Action 4, ‘Improving access for cities to EU integration funding’ (Urban Agenda for the EU, 2017, p. 14). According to interviews and informal conversations, together with CEMR and the DG Home, staff officers of the WGM&I engaged in the drafting of the ‘Recommendations for improving cities use of and access to EU funds for the integration of migrants and refugees in the new programming period’ (Urban Agenda for the EU, 2018). These recommendations advanced two main requests: an increase in EU funding for local integration policies; and the establishment of a ‘structured multi‐level governance framework’ based on the ‘principle of conditionality’, whereby in order to have access to funding national governments first had to establish partnership agreements with local authorities on implementation.

The recommendations of the Partnership were officially presented by the then mayor of Amsterdam to several European Commissioners and agreed on by the Civil Liberties, Justice and Home Affairs Committee of the European Parliament in its proposal for reform of the Asylum and Migrant Integration Fund regulations (European Parliament, 2019). However, my interviewees reported a much less supportive stance on the part of the European Council of Home Affairs Ministers, especially with respect to the principle of conditionality, and only mild engagement of the EC in supporting the reform of EU funding in this direction. As I observed during the final conference of the Cities Grow project (Milan, 7–8 November 2018), some members of Solidarity Cities were clearly putting pressure on the DG Home to support the introduction of the conditionality principle in EU funding, somewhat challenging the more accommodative stance prevailing among Eurocities executive directors and staff officers.

## IV Welcoming America

Analysis of the WA official website and of the many documents and the audio‐visual material published on it seems to converge in depicting a typical ‘American story’ (WA_int6): an NGO mobilizing grassroots initiatives around the key idea of building welcoming communities through horizontal networks and mutual knowledge between long‐term residents and new immigrants (see also Downs‐Karkos, 2011). The origins of WA were actually rooted in the experience of the Tennessee Immigrant and Refugee Rights Coalition (TIRRC), which was founded in Nashville in 2001 by David Lubell, ‘an accomplished social entrepreneur’ as he is described on the WA website. His ideas and discourse on ‘welcoming’ are at the very core of the gradual expansion of WA as a ‘movement of communities’:

At the beginning of the 2000s the climate in Nashiville was very tense. I started by mobilizing a coalition of neighbourhood associations and migrant groups, and in 2006 we were able to start a dialogue with the local government and to launch the Welcoming Tennessee initiative. The focus was really on local communities and on changing attitudes and improving the overall climate around migration. Welcoming Tennessee got noticed by other local coalitions and private foundations willing to support welcoming projects in their communities. We became a model from Nebraska to Massachusetts, and in 2009 WA was finally established as a non‐profit organization (WA_int2).

The horizontal agenda of the WA civil society activists, and more specifically of Lubell and the deputy directors, is well reflected in WA flagship initiatives, which show a prevailing network governance mode of policy‐making. On the one hand, WA has been able to incorporate under its umbrella independent networks like the Rust Belt Initiative and Global Detroit. Established in 2012 by 20 economic development coalitions – including non‐profit organizations, private companies and local authorities – to valorize contributions by immigrants to counter the demographic and economic downturn of the Midwest, the Rust Belt network formally joined WA in 2014. On the other hand, the network has always directly promoted new initiatives in the spirit of building partnerships between public and non‐public actors and horizontal exchanges between communities, like Welcoming Cities and Counties (WCC) and Welcoming International.

WCC was launched in June 2013 with the support of the German Marshall Fund to favour the sharing of innovative policies and develop new tools to improve ‘the quality of life and economic potential for of immigrants and non‐immigrants alike’. Following in these footsteps, with the help of ‘leading experts’ (that is, practitioners, academics, and business and civic leaders), in 2016 WA drafted The Welcoming Standard, a series of rigorous benchmarks and requirements' that communities have to meet in order to be certified as ‘welcoming’ (Welcoming America, 2016, p. 10). The certification process has to be initiated by a city or county government, but ‘partners, such as non‐profits, can contribute at any stage, including completing the application, contributing to the self‐assessment, and being consultants during the site visit’ (Welcoming America, 2016, p. 11). Furthermore, the assessment criteria consider collaboration and partnership to be the key strategy to build welcoming and cohesive communities. As for Welcoming International, this programme was formally established in 2015, always in collaboration with the German Marshall Fund. Thanks to financial support from various German and American foundations, three rounds of exchanges between cities on both sides of the Atlantic were organized between 2016 and 2018 engaging 30 municipalities. Since 2017, Welcoming International has been supporting welcoming initiatives in Australia, New Zealand and the UK.

Notwithstanding the clear focus of the WA leaders' policy agenda on horizontal, public/non‐public and city‐to‐city relations, the network was prominently engaged in MLG policy‐making processes initiated by the Obama Administration in 2014.

During the Obama Administration we started to have conversations on how the federal government could support the work that was already taking place at the community level. They were interested in understanding our work and the way they could direct more support to communities. There started to be meetings with different federal agencies […] In 2013 President Obama assigned the prestigious Champions of Change award to 10 leaders of WA. This recognition was important and opened up new opportunities for dialogue with our communities (WA_int1).

From this, contacts with the White House intensified, and more specifically with the Task Force for New Americans, an inter‐agency body created by the president in November 2014 to develop a ‘co‐ordinated federal strategy to better integrate immigrants into American communities’ (Task Force for New Americans, 2015, p. 3). The Task Force undertook extensive consultations with numerous stakeholders in both the public and private sectors and at all levels of government. A major outcome of this process was the Welcoming Communities Campaign, which was launched officially in April 2015 to support access by local communities to federal funding for immigrant integration and other resources. More specifically, this policy action established multi‐level governance relations between the Corporation for National and Community Service (part of the Federal Government), the YMCA, Catholic Charities and national refugee resettlement organizations, all engaged in supporting community work in WA member cities. The latter were able to get access to: (1) extra staff; (2) a webinar series on federal programmes and policies and best practices in cities and counties; and (3) a publication series entitled the Community Planning Toolkit (Reeves, 2015; Task Force for New Americans, 2015).

Relations with the White House stopped after the election of President Trump and the discontinuation of the Task Force. On the vertical dimension, relations seem to be primarily with members of Congress with the aim of lobbying for the Welcoming Communities approach to immigrant reception.

We have to row against the ideological momentum … continue our work in local communities, consolidate our approach and show that it works. We annually bring a delegation of our members from several parts of the US to meet members of Congress, and we present our work to both conservative Congress Representatives and Senators [...] Furthermore, we have informal relations with individual Senators because of the contacts of our members with the representatives of their districts (WA_int1).

The lack of significant links with national policymakers and the prevailing horizontal ambition of the network were also evident to me during the Pittsburgh annual convening I attended in May 2019. City governments, NGOs and civil society organization representatives made up the bulk of the participants in workshops and plenaries, while there were no official representatives of federal agencies with competence on migration.

## V Comparative Discussion. MLG at the Will of National Governments

The empirical narratives above provide a basis on which to explore the factors and mechanisms underlying the emergence of a CN‐MLG nexus on the contentious migration issue. To this end, I start by assessing the factors (EU institutions and problem pressure) and mechanisms (CN leadership) proposed in the existing literature, checking for similarities between the two cases in the processes leading to the operation of MLG‐like instances of policy‐making.

First of all, on the basis of accounts that emphasize the importance of EU institutions, we should expect to find WGM&I leadership considerably more involved in MLG policy‐making than WA. This is only partly true: there are some traces of MLG dialogue in the Integrating Cities Conferences, which, however, are public events with just symbolic value. In fact, the staff officers and city officials involved in the Integrating City Process appear to have been more interested in consolidating a direct partnership with the EC, while mayors have been more engaged in lobbying for direct access to EU funding. Second, regarding the problem pressure, my analysis reveals two key challenges that could have motivated CN leaders to pursue MLG policy‐making: the arrival and settlement of migrants in new destinations in the US and the refugee crisis in Europe. However, my findings do not seem to support such an expectation. In the case of WA, the Welcoming Cities and Counties initiative was promoted by CN civil society activists essentially in a perspective of strengthening their community‐based agenda with city‐to‐city policy learning and exchange of best practices. As for the Eurocities WGM&I, mayors – especially from southern Europe – launched the Solidarity Cities initiative primarily to voice their discontent with national and EU reception policies and lobby for the recognition of the key role of municipal governments in facing the emergency.

However, in both the EU and US contexts the new challenges mentioned above represented critical junctures for migration policy, offering a window of opportunity for MLG. My empirical accounts show that the initiative was taken from above, i.e. the Obama Administration and the Dutch Minister of the Interior in the context of the Urban Agenda for the EU. In the case of the Welcoming Communities Campaign, the Task Force provided the overall framework for establishing contacts between WA and relevant federal agencies. In that of the Partnership for the Inclusion of Migrants and Refugees, a key brokering role was played by the city of Amsterdam, even though in direct contact and in the context of an initiative launched by the Ministry of the Interior.

Hence, tracing causal processes leading to the engagement of CNs in MLG policy‐making on migration reveals that under the pressure of new migration challenges MLG was brought about primarily by ‘a move from above’. In the EU context, the city of Amsterdam, on the impulse of the mayor, was crucial in bringing actors together. However, in neither case did CNs play a decisive role in this initial phase, but became involved as representatives of the cities once MLG‐venues were established. Furthermore, evidence from my study shows that in the supranational EU context government authorities played a key gate‐keeping role not unlike that of the federal government in the US. The four representatives of national governments in the Partnership for the Inclusion of Migrants and Refugees not only showed limited interest in the process and participated only occasionally but in the end did not support its results, as is shown by the opposition of the Council of Ministers to the principle of conditionality reported by my interviewees.

These results cast some doubt on the enthusiasm for the role of migration CNs in bringing about participative and consensual policy‐making in MLG‐like arrangements (Penninx, 2015). Not only hierarchy but also national power seem to remain alive and kicking in instances of MLG migration policy‐making. However, this does not mean that CNs are simply spectators of quintessentially state‐led migration policy. In the EU, Eurocities has been able to establish an institutionalised partnership with the EC, Integrating Cities, which continued to work on integration issues even in the difficult context of the refugee crisis. In the US, WA has been able to spread the idea of the importance of building welcoming communities all over the country and, since 2011, also beyond.

## Conclusion

In this article, I have proposed an explorative analysis of the factors and mechanisms accounting for the genesis and involvement in MLG policy‐making of two migration CNs – that is, the WGM&I in the EU supranational system and WA in the US federal state.

Research evidence shows that, while migration CNs pursue different modes of policy‐making depending on their leaders' agendas, in both institutional contexts the emergence of instances of MLG has been highly conditioned by the will of national governments, and therefore has taken place in the shadow of hierarchy (Börzel, 2010).

It could be hypothesized that the activism of CNs at a the grassroots level in the US and in lobbying EC policies in the EU has played a role in the decisions of national governments to establish broad, vertical and horizontal MLG venues to face the complexity of challenges like arrivals in new destinations or the refugee crisis. However, the lack of similar arrangements during the Trump administration and the opposition of member states to the principle of conditionality seem to point to the fact that national governments remain key gatekeepers in the migration policy field and they can do this either by preventing the establishment of MLG policy‐making arrangements or by constraining their impact.

Hence, these results invite a more general reflection on how and to what extent the mobilization by sub‐national authorities can contribute to redefine state authority on a typical issue of state sovereignty such as migration. Rather than assuming a capacity of CNs to forge new policy‐making alliances beyond the nation‐state, by taking an explorative approach this study has unravelled the political work underlying CN mobilization in the migration policy field. As I demonstrate, instances of MLG policymaking, intended as a mode of governing based on coordination and collaboration between public and non‐public actors operating on different territorial scales, not only are rare but are also underpinned by deeply rooted power and political dynamics.

Whether these findings reflect a specificity of migration policy as a ‘least‐likely case’ for MLG or a more general limit of CN mobilization is still an open question.

Cross‐sectoral comparative research could further enrich our theoretical understanding of CN organizations and the political relations they are engaged in, contributing to throwing light on the nexus with MLG as a mode of policy‐making through which state authority is not simply dispersed but rather re‐defined vis‐à‐vis the challenges of globalization.

## List of Interviews

WGM&I_int1, Past Officer WGM&I (2004–2009), 26 July 2019.

WGM&I_int2, Project Manager, Eurocities Forum for Social Affairs, 10 July 2019, Brussels, Eurocities.

WGM&I_int3, Officer WGM&I, 4 April 2019, Brussels, Eurocities.

WGM&I_int4, Officer DG HOME, 4 April 2019, Brussels, European Commission.

WGM&I_int5, Representative local authorities in the Partnership for the Inclusion of Migrants and Refugees, Urban Agenda for the EU, 4 April 2019, Brussels.

WGM&I_int6, vice‐Mayor of a city member of the WGM&I, 23 July 2020, skype interview.

WGM&I_int7, city officer, member city of the WGM&I, 24 August 2020, skype interview.

WGM&I_int8, city officer, member city of the WGM&I and representative of cities in the Urban Agenda for the EU, 7 September 2020, skype interview.

WA_int1, Deputy Director, 24 July 2019, Washington, Welcoming America local office.

WA_int2, Deputy Director, 27 May 2019, Berlin, Welcoming America (Skype interview).

WA_int3, City Officer, Mayoral Migration Office, 18 July 2019, New York City.

WA_int4, National NGO member of WA, 16 May 2019, Pittsburgh.

WA_int5, County Officer member of WA, Migration Unit, 17 May 2019, Pittsburgh.

WA_int6, external expert engaged in WA activities between 2008 and 2010, 24 July 2020, skype interview.
